# *Drosophila* Innate Immunity Involves Multiple Signaling Pathways and Coordinated Communication Between Different Tissues

**DOI:** 10.3389/fimmu.2022.905370

**Published:** 2022-07-07

**Authors:** Shichao Yu, Fangzhou Luo, Yongyi Xu, Yan Zhang, Li Hua Jin

**Affiliations:** Department of Genetics, College of Life Sciences, Northeast Forestry University, Harbin, China

**Keywords:** *Drosophila*, innate immunity, immune response, signaling pathway, tissue communication

## Abstract

The innate immune response provides the first line of defense against invading pathogens, and immune disorders cause a variety of diseases. The fruit fly *Drosophila melanogaster* employs multiple innate immune reactions to resist infection. First, epithelial tissues function as physical barriers to prevent pathogen invasion. In addition, macrophage-like plasmatocytes eliminate intruders through phagocytosis, and lamellocytes encapsulate large particles, such as wasp eggs, that cannot be phagocytosed. Regarding humoral immune responses, the fat body, equivalent to the mammalian liver, secretes antimicrobial peptides into hemolymph, killing bacteria and fungi. *Drosophila* has been shown to be a powerful *in vivo* model for studying the mechanism of innate immunity and host-pathogen interactions because *Drosophila* and higher organisms share conserved signaling pathways and factors. Moreover, the ease with which *Drosophila* genetic and physiological characteristics can be manipulated prevents interference by adaptive immunity. In this review, we discuss the signaling pathways activated in *Drosophila* innate immunity, namely, the Toll, Imd, JNK, JAK/STAT pathways, and other factors, as well as relevant regulatory networks. We also review the mechanisms by which different tissues, including hemocytes, the fat body, the lymph gland, muscles, the gut and the brain coordinate innate immune responses. Furthermore, the latest studies in this field are outlined in this review. In summary, understanding the mechanism underlying innate immunity orchestration in *Drosophila* will help us better study human innate immunity-related diseases.

## Introduction

We live in an environment surrounded by different pathogens, including bacteria, fungi and viruses. Our immune system, which involves immunological organs, blood cells and other defense mechanisms, combat these invading pathogens. However, the worldwide spread of coronavirus disease 2019 (COVID-19) into a pandemic has suggested that infectious diseases are still major threats to human health (1). Therefore, discovering how organisms recognize and eliminate pathogens is an urgent goal. To study host-pathogen interactions, many *in vivo* and *in vitro* studies have been performed with murine models and mammalian cell lines, respectively (2, 3). In fact, the signaling pathways and biological processes associated with innate immune responses are highly conserved in *Drosophila* and vertebrates. For instance, nuclear factor-kappa B (NF-κB) signaling, phagocytosis and apoptosis are evident in the innate immunity of both vertebrates and invertebrates (4–8). Hence, it is possible to utilize insect models such as flies and nematodes to investigate pathogen and host interactions. In addition, the immune system of insects can be investigated relatively simply because they lack adaptive immunity, and genetic manipulation in insects is tractable (9–11).

The fruit fly *Drosophila melanogaster* lives in decaying organic matter. However, these flies are not infected by pathogens under this condition, largely because of their powerful immune defense system. As the first line of defense, *Drosophila* innate immunity comprises multiple strategies to fight against invading pathogens (12). First, the epithelial systems in the epidermis, trachea and gut provide physical barriers to obstruct the entry of bacteria and other pathogens. In addition, a local immune response follows pathogen attack at an epithelial site; for instance, insects produce antimicrobial peptides (AMPs) and reactive oxygen species (ROS) that enable the gut to combat oral infection (13). In addition, phagocytosis and encapsulation by plasmatocytes and lamellocytes, respectively, play important roles in the cellular responses of *Drosophila*. Plasmatocytes are macrophage-like hemocytes that can phagocytose pathogens (14, 15), whereas lamellocytes encapsulate large particles such as wasp eggs (16). Notably, lamellocytes are rare in healthy larvae, and they are differentiated upon immune challenge and in disadvantageous environments (17–19). As a third blood cell type, crystal cells are indispensable in wound healing, which is mediated by melanization (20). Furthermore, the fat body, functionally equivalent of the mammalian liver, is a vital immune tissue in *Drosophila*, playing a role in addition to metabolism. After infection, fat bodies secrete various AMPs into the hemolymph to kill invading microorganisms (21, 22). This process is the hallmark of the humoral immune response, also known as the systemic immune response. AMP production largely depends on two NF-κB-related signaling pathways: the Toll and immune deficiency (Imd) pathways (12). Interestingly, these two pathways show affinity for different pathogen types, with the Toll pathway more likely to respond to gram-positive bacteria and fungi and the Imd pathway primarily capable of responding to gram-negative bacteria (21). At the end of the 20 th century, the study of Toll signaling pathways in flies led to the identification of the mammalian Toll-like receptor (TLR), which makes a large contribution to the field of innate immunity (23). Thereafter, using *Drosophila* as a model to investigate the mechanism of innate immune responses has become increasingly popular, and host-pathogen interactions are largely understood because these signaling pathways are conversed between flies and humans, genetic tools are available and fly mutants are abundant. Moreover, through the *UAS/Gal4* system, the genetic expression of viral factors in pathogens of flies can be studied in infectious diseases and is a current research hotspot (24).

In this review article, we first describe the main components involved in *Drosophila* innate immunity, including immune tissues, cells, and signaling pathways. We also summarize tissue communication in terms of immune responses. Finally, we briefly explain the reasons that *Drosophila* is an ideal model to study innate immunity.

## Introduction to *Drosophila* Innate Immunity

**Figure 1:**
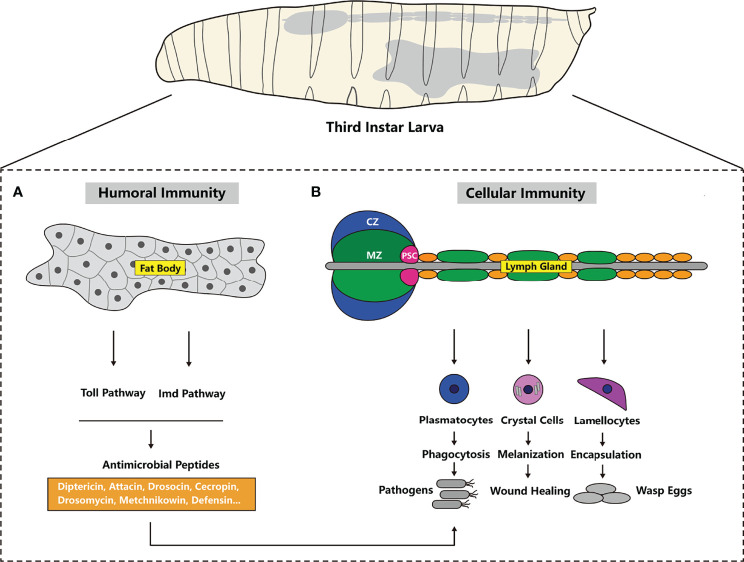
Overview of *Drosophila* host defenses. **(A)** The hallmark of humoral immunity (also known as the systemic immune response) is antimicrobial peptides (AMPs, i.e., Diptericin, Attacin and others) secretion by the fat body; this process is mainly modulated by the Toll and Imd pathways. **(B)** Cellular immunity depends on three types of hemocytes in *Drosophila*: plasmatocytes, crystal cells and lamellocytes. In addition the lymph gland is the main hematopoietic tissue in the larval stage. Plasmatocytes and lamellocytes can phagocytose and encapsulate pathogens, respectively, and crystal cells participate in wound healing through melanization.

*Drosophila* is an ideal model for studying innate immunity because these organisms do not produce an adaptive immune response. Various bacteria, fungi, viruses, parasitoid wasp eggs and aberrant host cells (wounded tissues, tumors, etc.) can induce a *Drosophila* immune response. *Drosophila* innate immunity can be classified into two kinds: humoral immunity involving fat bodies and hemolymph and cellular immunity mediated by immune cells (mostly hemocytes). In this review, we introduce humoral immunity executor AMPs, *Drosophila* hemocytes and the cellular immunity processes to which different kinds of hemocytes contribute (**Figure 1**).

### *Drosophila* Humoral Immunity Depends on AMPs

AMPs are small peptides that kill microbial cells (22, 25, 26). AMP expression is regulated by NFκB immune signals. When the immune signal is activated, AMPs are produced in the fat body and released into hemolymph (**Figure 1A**). Because AMPs are positively charged, microbes with a negatively charged cell membrane recruit AMPs to the hemolymph. Then, the AMPs can embed in the hydrophobic region of the microbe cell membrane and cause membrane destabilization and cell death (27). This wide-ranging process is called the systemic immune response. In addition, some susceptible tissues, such as the trachea, midgut, oviduct, spermatheca, ganglia and a subpopulation of hemocytes, produce AMPs in response to local infection (28, 29). Overall, the expression level of AMPs directly reflects the strength of the immune response.

Twenty AMP genes and another AMP-like gene have been identified in *Drosophila* thus far (**Figure 1A**) (22, 30). According to the structures of the peptides, 20 AMP genes have been classified into 7 families: Diptericin (Dipt or Dpt), Attacin (Att), Drosocin (Dro), Cecropin (Cec), Defensin (Def), Drosomycin (Drs) and Metchnikowin (Mtk). According to their targets, 7 AMP families have been classified into 3 categories: response to fungi (Drs and Mtk), gram-positive bacteria (Def) and gram-negative bacteria (Att, Cec, Dro and Dpt).

### *Drosophila* Cellular Immunity

#### *Drosophila* Immune Cells

Generally, *Drosophila* circulating hemocytes are considered to be immune cells because they play key roles in cellular immunity. Because of the higher quantity and variety of hemocytes in the larval stage, larval hemocytes are extensively studied (16). On the basis of morphological and cytochemical analyses, three types of hemocytes have been identified: plasmatocytes, crystal cells and lamellocytes (**Figure 1B**) (15, 16, 19, 31, 32). Plasmatocytes, which are round and small, account for the majority of circulating hemocytes (95%). The immunological function of plasmatocytes is phagocytosis of small pathogens, similar to mammalian macrophages/monocytes (**Figure 1B**) (15, 16, 32). Another 5% of the circulating hemocyte population consists of crystal cells that contain crystalline inclusions (15, 16, 19, 32). With prophenoloxidase (PPO) in crystalline inclusions, crystal cells participate in melanization (**Figure 1B**) (15, 19, 32–34). Lamellocytes can only be seen in larvae under immune challenge. Lamellocytes are large and flat, and they encapsulate large invading pathogens that plasmatocytes are not able to phagocytose, such as parasitic wasp eggs (**Figure 1B**) (15, 16, 19, 31, 32).

With advances in single-cell sequencing, some studies have identified subpopulations of the three classical types of hemocytes or have proposed new types on the basis of differentially expressed genes. For example, in 2020, Cattenoz et al. described 13 subpopulations of plasmatocytes (PL-0-PL-3 and 9 other subpopulations with specific molecular signatures), 1 subpopulation of crystal cells and 2 subpopulations of lamellocytes (LM-1 and LM-2) (35). In the 13 plasmatocyte should be subpopulations, PL-Rel, PL-vir1, PL-robo2, PL-Amp and PL-ImpL2 have been suggested to participate in specific immune responses, as indicated by a GO term enrichment analysis. The PL-Rel subpopulation expresses the transcription factors Toll and Imd. The marker of the PL-vir1 subpopulation is vir1, which responds to viral infection. GO term enrichment with the PL-robo2 subpopulation has been related to migration and phagocytosis. The PL-Amp subpopulation significantly expresses AMPs. Some markers of the PL-ImpL2 subpopulation indicate that this cluster creates a niche through which immune cell differentiation is regulated, similar to the posterior signaling center (PSC) of the lymph gland (the *Drosophila* hematopoietic organ during the larval stage), which is the hematopoietic niche (19, 32, 36, 37). The LM-1 and LM-2 subpopulations of lamellocytes represent mature lamellocytes and hemocytes in the plasmatocyte/lamellocyte intermediate state, respectively. Tattikota et al. identified 12 subpopulations of plasmatocytes (PM1-PM12), 2 subpopulations of crystal cells (CC1 and CC2) and 2 subpopulations of lamellocytes (LM1 and LM2) (38, 39). In the 12 plasmatocyte should be subpopulations, PM3-PM7 are considered immune-activated plasmatocytes. The PM3-PM5 subpopulations highly express several immune response-induced genes, such as metalloproteinase 1 (Mmp1) and immune-induced molecule 18 (IM18). PM6 and PM7 highly express AMPs, similar to the PL-AMPs described in the Pierre et al. study. CC1/CC2 and LM1/LM2 are crystal cells and lamellocytes in the intermediate/mature state, respectively. Fu et al. identified 4 subpopulations of plasmatocytes (Ppn^+^ PMs, CAH7^+^ PMs, Lsp^+^ PMs and reservoir PMs), crystal cells, lamellocytes and two novel hemocyte types: thanacytes and primocytes (40). Four plasmatocyte subpopulations play defined roles in the immune system. Although *Drosophila* hemocytes do not comprise a lymphoid lineage, thanacytes specifically expressing *CG30088* and *CG30090* are homologous to human GZMB and GZMH, which are highly expressed in natural killer cells and CD8^+^ T cells (40).

#### Phagocytosis Depends on Plasmatocytes

In addition to plasmatocytes, cells of various types have been found to engulf particles such as pathogenic microbes and apoptotic cells (41, 42), and the phagocytosis process is similar between these cell types and plasmatocytes. However, as professional phagocytes, plasmatocytes take up more particles with greater efficiency than other cell types. Extracellular phagocytosis-facilitating processes include opsonization and recognition. Opsonins bind to microbes, making them easier for phagocytes to engulf. Opsonins are required for opsonization, which occurs in the humoral environment (43). The thioester-containing protein family has been reported to be an opsonin in *Drosophila* (44), and recognition of ligands on particles and receptors on plasmatocytes is triggered. Various particles and molecules on the surface of particles, including bacterial peptidoglycans (PGNs), lipopolysaccharides (LPSs), fungal β-1, 3 glucans and phosphatidylserine, which are exposed to the outer cell membrane in apoptotic cells, can be recognized by phagocytic receptors. Most phagocytic receptors overlap with molecular markers of plasmatocytes, including Croquemort (Crq) (45), Nimrod C-type proteins NimC1 (46, 47) and Eater (19, 32, 48, 49). In addition, two subunits of Integrin, Integrin αPS3 and Integrin βν, have been reported to mediate phagocytosis of apoptotic cells and bacteria, indicating that these subunits may be phagocytic receptors (50, 51). Therefore, plasmatocyte uptake of particles depends on the dynamic remodeling of the plasma membrane.

Intracellular events of phagocytosis include phagosome formation, maturation and degradation. After particle internalization, phagosomes form. Then, phagosomes mature through fusion with endosomes, which endows phagosomes with bactericidal ability. Finally, upon fusion with lysosomes, phagosomes are degraded by hydrolases in the lysosomes, completing the clearance of the particles engulfed by the phagosomes (52).

#### Antitumor Effects of Plasmatocytes

**Figure 2:**
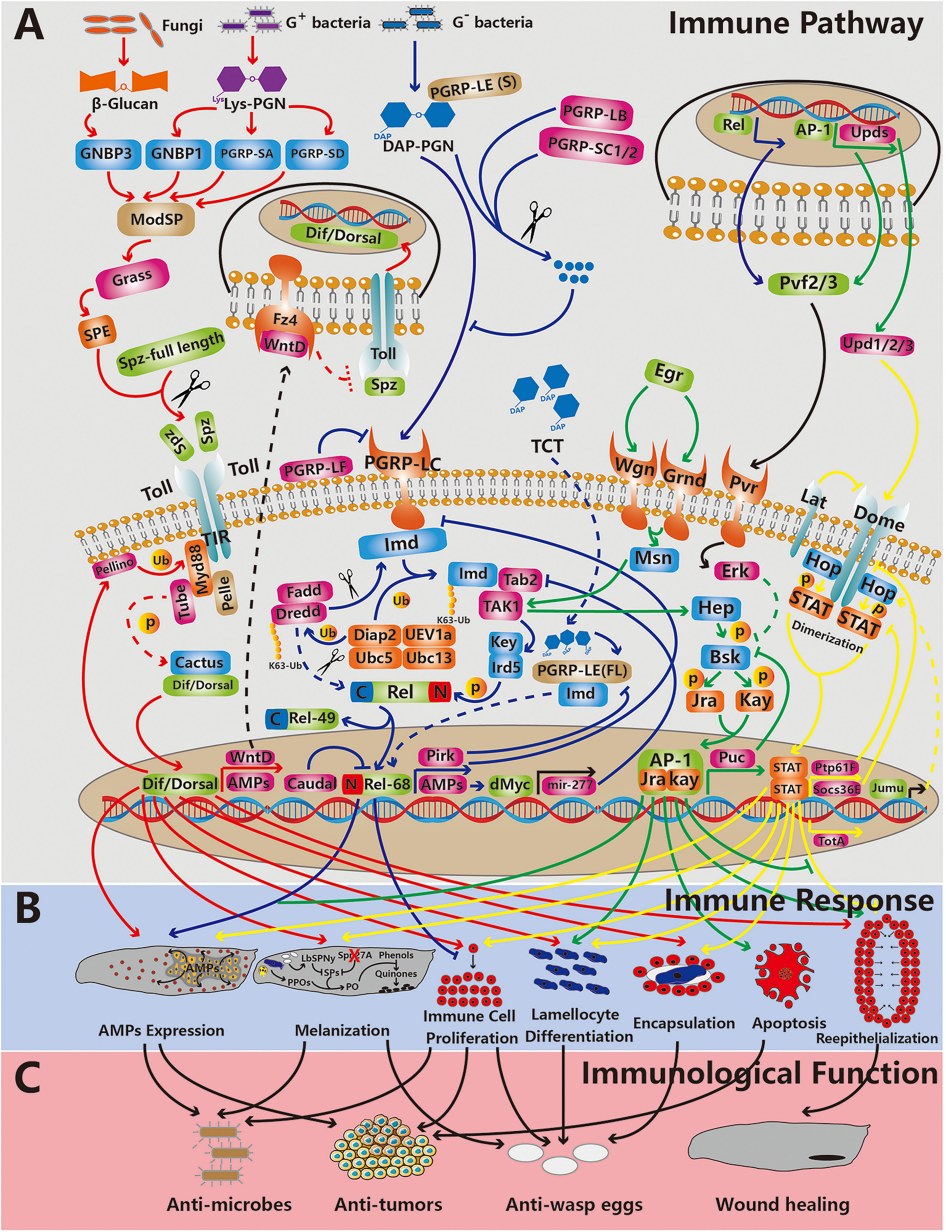
The relationship between main immune pathways, immune responses and immunological functions in *Drosophila* innate immunity. From top to bottom, the three rows represent the immune pathway (the Toll, Imd, JNK and JAK/STAT pathways are depicted,from left to right) **(A)**, the immune response **(B)** and immunological functions **(C)**. The red, blue, green and yellow lines indicate that the processes are related to the Toll, Imd, JNK and JAK/STAT pathways, respectively. The black lines indicate that the processes are related to other pathways. The dotted lines represent processes waiting to be confirmed. The arrows and “T” indicate promoting and inhibitory effects, respectively. The red cross indicates depletion. The scissors represent cleavage. Abbreviation: p, phosphorylation; Ub, ubiquitination; (s), short form; and (f), full length.

Recent studies have shown that plasmatocytes are antitumor immune cells in *Drosophila* because: 1. these cells are recruited and specifically associated with tumors (53, 54), 2. their proliferation is activated in flies with tumors (53), 3. they express *Drosophila* tumor necrosis factor (TNF) and Eiger (Egr) to induce tumor cell apoptosis (55, 56), 4. they take up AMPs to induce tumor apoptosis (54, 57), and 5. they phagocytose tumor cell fragments (**Figures 2A–C**) (55).

Plasmatocytes are recruited by tissue basement membrane damage caused by tumors (53). Plasmatocytes are associated with tumors and express TNFs to induce tumor cell apoptosis (55, 56). In addition, tissues burdened with tumors secrete Upd cytokines and Pvf1 into the hemolymph, which causes an increase in the number of circulating plasmatocytes (53, 56). Furthermore, the expression level of the Toll signaling ligand Spätzle (Spz) in circulating plasmatocytes is elevated. Toll signaling in the fat body is activated, which results in increased AMP levels (**Figures 2A, B**) (56). Therefore, AMPs are taken up and transported to tumors by plasmatocytes (54). In the presence of Egr, which is expressed during the immune response, tumors are sensitive to AMPs, which means that AMPs induce tumor apoptosis facilitated by Egr (**Figures 2B, C**) (57). In addition, plasmatocytes can phagocytose tumor cell fragments, which might be debris from apoptotic cells (55).

#### Cell Encapsulation of Pathogens

Parasitoid wasp infection is a health threat to larvae, in addition to microbial pathogens. Because parasitoid wasp eggs are too large to be phagocytosed by plasmatocytes, encapsulation of these eggs by lamellocytes to sequestrate and eliminate themis a key immune response (17, 58). Upon parasitoid wasp infection, immune signaling is activated, such as through the Toll and Janus kinase (JAK)/signal transducer and activator of transcription (STAT) signaling pathways; these pathways induce lamellocyte generation in both circulating hemocytes and the lymph gland (32). In a cell trajectory analysis with single-cell sequencing, lamellocytes were found to be derived in two ways in the lymph gland (59): 1. hematopoietic progenitor differentiation into lamellocytes (60–63) and 2. plasmatocyte transdifferentiation into lamellocytes (64, 65). However, there is only one way for circulating hemocytes to become lamellocytes; plasmatocytes are transdifferentiated into lamellocytes (**Figure 2B**) (35, 39, 66). The lymph gland dissociates several hours after infection and then releases lamellocytes into the circulatory system (62, 63).

Upon parasitoid wasp infection, plasmatocytes are recruited and distributed on the surface of the wasp eggs (67). Then, plasmatocytes transdifferentiate, and lamellocytes are then recruited by plasmatocyte-coated eggs to initiate encapsulation (**Figure 2B**) (65). Ao et al. noted that C-type lectins DL2 and DL3 on the surface of *Drosophila* hemocytes are recognition receptors of agarose bead encapsulation (68). However, the recognition receptors on hemocytes and molecules on the surface of parasitoid wasp eggs require further study. During encapsulation lamellocytes organize to form multilayered capsules to sequester pathogens. Proper localization of Integrin-β to the plasma membrane of lamellocytes is required for this process (33, 69). Pathogens are thus eliminated by melanization, which depends on the actions of both lamellocytes and crystal cells (**Figures 2B, C**) (details in 2.2.5).

Naoaki et al. showed that one kind of encapsulation, called phagocytic encapsulation, depends on enlarged plasmatocyte phagocytosis (70). Overexpression of *Drosophila* p38 MAPK kinase plasmatocytes in *Salmonella*-infected flies increased 3 to 4-fold compared to uninfected flies, and the flies are filled with phagocytosed *Salmonella* in the late stage of infection. Phagocytic encapsulation regulated by *Drosophila* p38 MAPK kinase can sequester pathogens and increase the survival rate after infection.

#### Melanization by Crystal Cells

Melanization contributes to blood coagulation, wound healing and encapsulation. Melanization involves phenol oxidation to quinones, which polymerize to generate melanin. Phenol oxidation is required for the generation of phenol oxidase (PO), which is derived from the proteolytic cleavage of PPO. Upon melanization, PPO is released into the hemolymph by crystal cell rupture and proteolytic cleavage by serine protease (SP), which is activated by pathogens and wounds (**Figure 2B**). Thus, melanization is a combined cellular and humoral immune response.

Pyroptosis is a programmed cell death in mammals. Pyroptosis involves cell membrane swelling, causing rupture and cytosolic content release, which induces inflammation (71). *Drosophila* crystal cell rupture is similar to pyroptosis. Recently, Dziedziech et al. showed *Drosophila* crystal cell rupture as a type of proto-pyroptosis, an ancient form of pyroptosis, that is dependent on Caspase activity (72). Previous studies have indicated that crystal cell rupture is required for JNK activation by Egr (73) and ROS (74). Dziedziech et al. first proposed that crystal cells are activated by JNK signaling, while ROS production promotes JNK signaling. Subsequently, cell membranes begin to swell, and the Caspase cascade induces crystal cell rupture (72).

Three *PPO* genes have been identified in the *Drosophila* genome: *PPO1*, *PPO2* and *PPO3*. *PPO1* and *PPO2* are mainly expressed in crystal cells. *PPO3* is mainly expressed in lamellocytes (33). *PPO3* differs from *PPO1* and *PPO2* in that only *PPO3* overexpression can induce spontaneous melanization in the absence of pathogens. This function implies that the zymogen form of PPO3, but not that of PPO1 or PPO2, is enzymatically active independent of SPs. Moreover, PPO3 is required for melanization in the *hop^tum-l^* mutant, in which JAK/STAT is activated and many lamellocytes are in circulation (75). However, to reveal the detailed processes by which lamellocytes participate in melanization and PPO3 is released, further study is required.

The N-termini of the PPOs are cleaved by SP, which results in the activation of POs. Approximately 150 genes encoding SPs have been found in the *Drosophila* genome (76). Three SPs have been identified as PPO activators in hemolymph: MP1, MP2 (also called Sp7 or PAE1) and Hayan (77–79). Dudzic et al. pointed out that two distinct SP pathways induce melanization. Hayan activates both PPO1 and PPO2, specifically inducing the melanization reaction, which blackens wound sites. In addition, MP2 activates only PPO1, inducing alternative melanization and thus killing pathogens such as *Staphylococcus aureus* (80). In addition, MP1 is required for melanization in response to both bacterial and fungal infection. MP2 mainly participates in melanization in fungal infection. These differences imply that MP1 is a common downstream SP that activates PPOs through different melanization cascades activated by bacteria or fungi exposure. This means that MP1 acts downstream of MP2, and MP2 cannot directly activate MP1 *in vitro* (81).

Constitutive melanization is harmful to flies and even causes death. The SP inhibitor serpin can also regulate melanization. Three serpins have been shown to inhibit excessive melanization in *Drosophila*: Spn27A, Spn28D and Spn77Ba (82–84). Upon infection, Spn27A is depleted from the hemolymph and activates PO (85), causing melanization. Spn77Ba is an inhibitor of the MP1 and MP2 protease cascades in *Drosophila* trachea (84). In addition, the venom of the parasitoid wasp *Leptopilina boulardi* contains a serpin, LbSPNy, which allows their eggs to escape melanization in *Drosophila* (**Figure 2B**) (86).

## *Drosophila* Immune Signaling Pathways

Activated immune signals induce the expression of immune response genes, which comprise the core of *Drosophila* innate immunity. Typically, immune signals are initiated by recognition between pathogen-associated molecular patterns (PAMPs) on pathogens and pattern recognition receptors on host cells. The PAMP-induced signal transduction is mediated by adaptor proteins in the host cell. Activated kinases, proteases and ubiquitin ligases contribute to biochemical modifications of the core transcription factors in immune signaling pathways. Ultimately, the modified transcription factors are translocated to the nucleus and promote immune-related genes in response to immune challenge. In this section, we summarize the main 4 immune signaling pathways in *Drosophila*: NFκB/Toll, NFκB/Imd, JAK/STAT and JNK (**Figure 2A**).

### The Toll Pathway

The Toll pathway, which is part of the NFκB pathway, was first identified in the dorsal-ventral patterning of embryos. NFκB family transcription factors regulate immune-responsive gene expression to defend the host. There are 2 NFκB family transcription factors in the Toll pathway: Dorsal and Dif. Toll is mainly activated by gram-positive bacteria and fungi. The Toll pathway contributes to both humoral immunity and cellular immunity. In addition, the Toll pathway is required for melanization and epidermal wound repair during the late embryonic stage (**Figure 2C**).

#### Overview of the Toll Pathway

Important extracellular processes of the Toll pathway are recognition, SP cascade and the cleavage of ligand Spz. The recognition molecules on gram-positive bacteria and fungi are lysine (Lys)-type PGNs and β-glucan, respectively. The recognition factors gram-negative binding protein (GNBP1), GNBP3, PGN recognition protein (PGRP)-SA and PGRP-SD react with these recognition molecules on the surface of microbes (87–90). GNBP3 specifically participates in yeast recognition (90). The other 3 recognition factors mainly recognize Lys-type PGNs on gram-positive bacteria. In addition, the recognition of diaminopimelic acid (DAP)-type PGNs on gram-negative bacteria is mediated by PGRP-SD, which induces Toll pathway activation (91). The signals emitted through these recognition processes are integrated by the modular SP ModSP (92). Gram-positive-specific SP (Grass) and 4 other SPs, Spirit, Spheroide, Sphinx1 and Sphinx2, localize downstream of ModSP, which induces Spätzle-processing enzyme (SPE) activation (93, 94). Ultimately, a ligand in the Toll pathway, Spz, is cleaved by activated SPE and binds with the receptor Toll (**Figure 2A**) (95).

Important intracellular processes in the Toll pathway include the degradation of Cactus and nuclear translocation of Dorsal and Dif. Under normal conditions, the NFκB family transcription factors Dorsal and Dif are bound to Cactus and primed for nuclear translocation (23, 96). Upon activation, Spz binds to the Toll receptor, and the Toll receptor intracellular Toll/Interleukin-1 receptor (TIR) domain interacts with the adaptor protein MyD88, which forms a complex with the kinase Pelle and the adaptor protein Tube that can phosphorylate and degrade Cactus (**Figure 2A**) (97–100). Dorsal and Dif are then translocated to the nucleus, which promotes immune-related gene expression.

Negative feedback loops maintain hemostasis of the Toll pathway. The Wnt inhibitor of Dorsal (WntD) is a target in the Toll pathway and a feedback inhibitor of the Toll pathway in both developmental processes and immune responses to septic infection (101). The expression of *dipt* remains high following infection in *wntD*-null mutants (101). Rahimi et al. showed that WntD is secreted and associated with its receptor Frizzled4 (Fz4), which blocks the Toll extracellular domain in the dorsal-ventral patterning of embryos (102). However, whether this mechanism is the same as that mediating the WntD inhibitory effect on the Toll pathway during the immune response remains to be confirmed. Another negative regulator of the Toll pathway is the ubiquitin E3 ligase Pellino (103). Pellino was initially thought to be a positive regulator of the Toll pathway because the AMP Drs expression level in Pellino-null mutants is lower after *Micrococcusluteus* infection (104). However, additional data indicated that Pellino is a negative regulator of the Toll pathway (105). Ji et al. indicated that after the Toll pathway is activated, Pellino is recruited by MyD88 and accumulates on the plasma membrane. MyD88 is then ubiquitinated and degraded by Pellino, inducing negative regulation of the Toll pathway (**Figure 2A**) (105).

#### Immunological Function of the Toll Pathway

The Toll pathway directly promotes the expression of genes encoding AMPs, including *drs*, *atta* and *mtk*, which play key roles in *Drosophila* humoral immunity (see section 2.1) (22). With respect to cell immunity, Toll contributes to immune cell survival and proliferation, lamellocyte differentiation and encapsulation (**Figures 2B, C**). Matova et al. found microbes in immune cells of the *Dif*-/*dorsal*-double-null mutant. Moreover, more dead immune cells were found in this double-null mutant (106). These findings indicated that Dif and Dorsal in immune cells autonomously regulate immune cell number and survival through the NFκB family transcription factor target DIAP1, which is related to apoptosis (107). In addition, a significant increase in immune cell density was found in a gain-of-function Toll receptor mutant (*Toll^10b^*) and other constitutively activated Toll mutants (108, 109). The activated Toll pathway causes lamellocyte differentiation of hemocytesin the circulatory system (110, 111) and lymph gland (112). Similarly, upon wasp eggs infection, Toll pathway activation in the niche induces lamellocytes differentiation and dispersion of the lymph gland (63). Sorrentino et al. found that the encapsulation capacity is reduced in a mutant with a Toll pathway component deleted (108). These studies showed that the Toll pathway is crucial to the immune response to parasitoid wasp infection.

The Toll pathway participates in a complicated interaction with melanization. In 2002, Ligoxygakis et al. showed that the depletion of serpin Spn27A in hemolymph depends on the Toll pathway, which induced a melanization response to infection (**Figures 2A, B**) (85). In 2004, Zettervall et al. found melanotic nodules in the *Toll^10b^* mutant (109). Later, in 2014, Schmid et al. found that melanotic nodule formation requires only Toll activation in fat bodies (113). However, in 2019, Dudzic et al. found that the extracellular processes involved in Toll pathway signaling and PPO-activated melanization both require the SP Hayan. Hayan localizes downstream of Grass and contributes to SPE activation (80). Interestingly, small melanized spots caused by epidermal defects are found in the *Dif-/dorsal*-double-null mutant (107). This finding implies that the Toll pathway is dispensable for melanization. However, remodeling E-cadherin, a component in adherens junctions, in a wound site repair is required for Toll pathway activation in the epidermis (114). This finding implies that the Toll pathway is essential for epidermal defect repair, which might explain the formation of melanized spots in the *Dif-/dorsal*-double-null mutant (**Figures 2B, C**).

### The Imd Pathway

The Imd pathway is activated by the nuclear translocation of another NFκB transcription factor, Relish (Rel), which promotes the expression of most genes encoding AMPs. The Imd pathway is indispensable for *Drosophila* resistance to microbes.

#### Overview of the Imd Pathway

The recognition of PGNs on the surface of a microbe is the first step of Imd pathway activation. DAP-type PGNs on gram-negative bacteria and a few gram-positive bacteria, such as *Bacillus* spp., are recognized by PGN recognition proteins (PGRPs) (115, 116). According to transcript size, *Drosophila* PGRPs are classified into the short (S) and long (L) form. PGRP-LC is the principal transmembrane receptor in the Imd pathway (117, 118). In addition, PGRP-LE is classified into the short and full-length form; the short form facilitates PGRP-LC action (119, 120). The short form of PGRP-LE is secreted into hemolymph and binds PGNs that are subsequently recognized by PGRP-LC (121). Full-length PGRP-LE localizes to the cytoplasm and recognizes monomeric PGN (also called tracheal cytotoxin or TCT) fragments, which gain access to a cell (122, 123). Furthermore, PGRP-LE that localizes in the cytoplasm can induce Imd pathway activation independent of PGRP-LC (119–121). Although PGRP-LF is associated with the membrane, it cannot bind PGNs. In addition, through its interaction with PGRP-LC, PGRP-LF is a negative regulator of Imd signaling (**Figure 2A**) (124–126).

The rate-limiting step in Imd pathway activation is the nuclear translocation of Rel. The phosphorylation of multiple sites in the Rel N-terminus is required for Rel activation. In addition, the C-terminus of Rel is an inhibitor of nuclear translocation because it can mask the nuclear localization signal in the N-terminus and inhibit Relish dimerization. Therefore, the nuclear translocation of Rel requires not only phosphorylation of the N-terminus but also cleavage of the C-terminus. The IκB kinase complex composed of Ird5 and Kenny is thought to phosphorylate the Rel N-terminus (127). The caspase Dredd can cleave the Rel C-terminus (Rel-49) and N-terminal (Rel-68) *in vitro* (128). Therefore, Rel-49 is maintained in the cytoplasm, and Rel-68 is translocated into the nucleus to promote immune-related gene expression (**Figure 2A**) (128, 129).

Upon PGN binding with a receptor in the Imd pathway, a complex composed of Imd, Fadd, and Dredd is formed (117, 130, 131). The E3 ubiquitin ligase Diap2 associates with the E2 ubiquitin conjugating enzymes UEV1a, Ubc13 and Ubc5 to activate Dredd through ubiquitination (132, 133). Then, Imd is cleaved by activated Dredd to generate a Diap2-binding site, where K63-polyubiquitin chains are conjugated Imd (133, 134). K63-ubiquitination of Imd induces the recruitment and activation of a complex composed of Tab2 and TAK1, which phosphorylates and thus activates the Ird5/Kenny complex (127, 135–137). Finally, after phosphorylation and cleavage, Rel-68 is translocated to the nucleus (**Figure 2A**).

Negative feedback loops are needed to restore Imd pathway homeostasis after infection. Pirk, a target of the Imd pathway, interacts with PGRP-LC, PGRP-LE and Imd to inhibit signal transduction (138). Li et al. indicated that the transcription factor dMyc contributes to negative feedback loops that maintain Imd pathway homeostasis. Dipt, a target AMP in the Imd pathway, may promote *dmyc* expression. dmyc promotes the expression of the microRNA *mir-277*, which inhibits Imd and Tab2 expression (**Figure 2A**) (139).

#### Immunological Function of the Imd Pathway

Activation of the Imd pathway in the fat body promotes AMP production, which is released into hemolymph in response to systemic infection (**Figures 2A, B**). Because TCT in oral bacterial infection is a small molecule that can cross the gut barrier and enter hemolymph (140), the Imd signaling pathway in guts and hemocytes contributes to immune signaling to fat body cells. *dro* expression is downregulated by the elimination of adult fly hemocytes *via* apoptosis or knockdown of an Imd pathway component in flies injected with the gram-negative bacterium *E. coli* (141). The signal emitted from hemocytes to fat bodies is unclear. However, intestinal local infection by *Erwinia carotovora* induces a systemic immune response in larval fat bodies; hemocytes play a key role in this process (142). As mentioned above, the Toll pathway in the niche of lymph glands contributes to cellular immunity through the promotion of lamellocyte differentiation and dispersion of the lymph gland during systemic infection (63). Rel is inhibited upon bacterial infection, which disrupts Hedgehog signaling and thus hematopoietic progenitor maintenance (36). Moreover, hematopoietic progenitors differentiate into plasmatocytes but not lamellocytes during the cellular immune response (143).

As the digestive tract, the *Drosophila* gut contains various microorganisms. The Imd signaling pathway in the gut interacts with local commensal bacteria. On the one hand, commensal bacteria activate the Imd signaling pathway to a basal level in response to pathogenic bacteria exposure. Ryu et al. found that in the flies grown under germ-freeconditions without commensal bacteria in their guts, Rel did not localize to the nucleus (144). Later, Glittenberg et al. showed that germ-free flies are more susceptible to *Candida albicans* infection (145). These studies indicated that the presence of commensal bacteria can induce chronic basal activation of the Imd signaling pathway in response to pathogen infection. On the other hand, negative feedback loops maintain immune tolerance in the case of commensal bacterial death. The homeobox transcription factor Caudal is thought to be a negative regulator of the Imd signaling pathway (**Figure 2A**). Knocking down *caudal* expression disrupts the commensal community structurein the gut (144). Bosco-Drayon et al. showed that PGRP-LB and PGRP-SC1/2 expression depends on PGRP-LE sensing PGNs of commensal *Lactobacillus plantarum* (146). Moreover, PGRP-LB and PGRP-SC1/2 are thought to be negative regulators of the Imd signaling pathway, which they mediate through their amidase activity, leading to PGN elimination in case it has been recognized by PGRP-LC (147). In addition, Guo et al. found that overexpression of PGRP-SC2 in the gut reduces the commensal dysbiosis caused by aging (**Figure 2A**) (148).

### The JAK/STAT Pathway

The JAK/STAT signaling pathway regulates multiple immune processes. The JAK/STAT pathway participates in the systemic immune response to tumors, epidermal wounds, mechanical stress and parasitoid wasp infection. In addition, the activation of the JAK/STAT pathway promotes the proliferation of *Drosophila* intestinal stem cells (ISCs), which constitute a unique cell type with the ability to undergo mitosis in the gut (149). Therefore, the JAK/STAT pathway is triggered by a local immune response in the *Drosophila* gut and promotes epithelial repair.

#### Overview of the JAK/STAT Pathway

Similar to the other pathways described above, the nuclear translocation of STAT92E signifies that the JAK/STAT pathway has been activated. In *Drosophila*, 3 Unpaired (Upd) family cytokines, Upd1, Upd2 and Upd3, are ligands of the JAK/STAT pathway (150–154). These ligands can bind to Domeless (Dome), a unique receptor in the *Drosophila* JAK/STAT pathway (155, 156). A JAK unique to *Drosophila* is Hopscotch (Hop), which is constitutively associated with the intracellular Dome region (157, 158). Then, Dome dimerization induces hop activation. The activated Hop undergoes autophosphorylation and phosphorylates specific tyrosine residues in the intracellular region of Dome where STAT92E docks (159, 160). In addition, activated Hop phosphorylates STAT92E, which induces STAT92E dimerization and nuclear translocation (**Figure 2A**) (154). JAK/STAT pathway regulators are important to multiple biological processes. The structure of Latran (Lat), which is associated with the receptor complex, is similar to that of Dome. Because Lat lacks an intracellular site where STAT92E can dock, it may inhibit JAK/STAT signal transduction (161, 162). Recently, Jumeau (Jumu) was shown to be required for the nuclear translocation of STAT92E within *Drosophila* hemocytes and within both the cortical zone (CZ) and medullary zone (MZ) of the lymph gland. As a member of the *Drosophila* Forkhead transcription factor family, Jumu may regulate the expression of *hop*, affecting the phosphorylation of STAT92E (**Figure 2A**) (163).

Undoubtedly, negative feedback loops are needed to restore the JAK/STAT pathway. The suppressor of cytokine signaling protein 36E (Socs36E) is a target of JAK/STAT (164, 165). Socs36E not only can directly interact with Dome and inhibit Dome phosphorylation by Hop but can also indirectly affect the endocytosis of Dome (166, 167). Another target of the JAK/STAT pathway is protein tyrosine phosphatase 61F (Ptp61F), which has been inferred to inactivate phosphorylated Hop or STAT92E (**Figure 2A**) (168, 169).

#### Immunological Function of the JAK/STAT Pathway

Activation of the JAK/STAT pathway in fat bodies promotes the expression of Turandot A (TotA) and releases TotA into hemolymph, which enhances resistance to stress, such as bacterial infection, heat shock and ultraviolet light exposure (170). In addition, the JAK/STAT pathway contributes mainly to cellular immunity, such as plasmatocyte and lamellocyte differentiation, under stress and intestinal epithelium renewal after incurring cell damage (**Figures 2B, C**). The JAK/STAT signaling response to parasitoid wasp infection in the lymph gland is heterogeneous. The hematopoietic progenitors, which are located in the inner region in the anterior lobe of the lymph gland (known as MZ) and nearly the whole posterior lobe of the lymph gland, highly express the JAK/STAT pathway receptor Dome (19, 32). Moreover, JAK/STAT has been proven to promote the maintenance of these hematopoietic progenitors (61, 171). Interestingly, the JAK/STAT response pattern to parasitoid wasp infection is different between progenitors in the MZ and posterior lobe of the lymph gland. Upon parasitoid wasp infection, the activity of the JAK/STAT pathway in the progenitors of the MZ is downregulated by Upd3 and Lat, which promotes progenitor differentiation into lamellocytes and plasmatocytes (162). In contrast, the JAK/STAT pathway in the progenitors in the posterior lobe of the lymph gland is activated during differentiation, and therefore, these cells are in reserve, primed to guarantee the appropriate quantity of hematopoietic progenitors during the third larval stage (172). In addition, aberrant activation of the JAK/STAT pathway in mature hemocytes, which localize to the outer region of the anterior lobe in the lymph gland (known as CZ), causes lymph gland hyperplasia and lamellocyte generation independent of parasitoid wasp infection (173). Minakhina et al. indicated that the JAK/STAT pathway in the CZ promotes the cell autonomous and non-cell autonomous differentiation of plasmatocytes and lamellocytes, respectively. Moreover, Pannier (downstream of JAK/STAT) is essential for plasmatocyte differentiation (174). Tokusumi showed that mechanical stress caused by squeezing induces JAK/STAT activation in multiple tissues, including in the CZ of the lymph gland, which promotes lamellocyte formation (175). In addition, aberrant activation of the JAK/STAT pathway causes melanotic nodule formation mediated by lamellocytes (176, 177). The ingestion of pathogenic bacteria damages the intestinal epithelium by the toxins released from the bacteria and from the ROS generated by the host immune response (178). Under this condition, Upd2 and Upd3 secreted from enterocytes in the gut promote the proliferation of ISCs to repair the damaged intestinal epithelium (179).

Upd family cytokines released from circulating hemocytes activate the JAK/STAT pathway in other tissues during the systemic immune response. Upon septic injury, Upd2 and Upd3, especially Upd3, are secreted from circulating hemocytes and activate the JAK/STAT pathway in fat body and gut cells. Agaisse et al. showed that Upd3 released from hemocytes causes JAK/STAT pathway activation in fat bodies upon septic injury, which promoted the expression of *TotA* (180). Chakrabarti et al. indicated that wounds caused by septic injection activate p-JNK signaling in hemocytes, which causes Upd2 and Upd3 release from hemocytes. These Upd family cytokines promote intestinal epithelium renewal and resistance to bacterial infection (181). In contrast, proliferation of circulating hemocytes is promoted by the cytokine Upd3 secreted by tumors (**Figures 2B, C**) (53).

### The JNK Pathway

The JNK signaling pathway is a part of the mitogen-activated protein kinase (MAPK) pathway. The JNK pathway senses stress, such as that caused by ROS, ultraviolet light exposure, DNA damage, bacterial infection and wounding.

#### Overview of the JNK Pathway

The *Drosophila* TNF ligand Egr (182, 183) binds to two TNF receptors, Wengen (Wgn) (184) and Grindelwald (Grnd) (185), which activate the JNK pathway kinase cascade. The *Drosophila* JNK pathway kinase cascade is composed of Msn (JNKKKK), TAK1 (JNKKK), Hep (JNKK) and Bsk (JNK). In addition, Slipper (Slpr) can act as a JNKKK during dorsal closure (186). These components of the JNK pathway kinase cascade are phosphorylated and activated gradually. Finally, Jra (also called dJun) and Kay (also called dFos) are phosphorylated by activated JNK and form a transcription factor heterodimer called AP-1 (187, 188). AP-1 promotes the expression of target genes that contribute to multiple biological processes, such as the serine/threonine protein phosphatase *puckered* (*puc*) (189, 190), the matrix metalloproteinases *mmp1/2* (191–194) and Upd family cytokines (**Figure 2A**) (53, 195).

The negative feedback loop of the *Drosophila* JNK pathway depends on the target gene *puc*, which dephosphorylates Bsk (189, 190). The Imd pathway activates the JNK pathway through activated TAK1. In addition, a common negative feedback loop through PDGF- and VEGF-related receptors Pvr and Erk connect the JNK and Imd pathways. The JNK pathway together with Imd promotes the expression of PDGF- and VEGF-related ligands Pvf2 and Pvf3. Both ligands activate Pvr, which inhibits Bsk phosphorylation through Erk. Moreover, Pvr is an inhibitor of Rel phosphorylation (**Figure 2A**) (196).

#### Immunological Function of the JNK Pathway

In humoral immunity, the Imd pathway coordinates with the JNK pathway to generate AMP (**Figure 2B**). Kallio et al. indicated that *hep*, *kay* and *msn* expression knockdown decreases Att expression levels upon infection *in vitro* (197). Delaney et al. showed that *puc* overexpression in fat body cells, the *bsk*-null mutant and the *jun*-null mutant block AMP gene expression (198). With respect to cellular immunity, the JNK pathway promotes lamellocyte differentiation (**Figure 2B**). The expression of constitutively active *hep* induces the formation of lamellocytes in circulating hemocytes and melanotic masses (109, 199). The number of lamellocytes is changed by aberrant JAK/STAT pathway activation in circulating hemocytes and can be reduced by *kay*-null mutation (199). These findings indicate that JAK/STAT activation induces lamellocyte differentiation in a partially JNK pathway-dependent manner. In addition, Upd family cytokines, which are the target of JNK pathway, are secreted by enterocytes, promoting proliferation of *Drosophila* ISCs during the repair of epithelial damage caused by microbes (195). The JNK pathway plays a dual role in tumors (200). On the one hand, the JNK pathway plays an antitumorigenic role through tumor apoptosis and immune cell recruitment. As described in section *Antitumor Effects of Plasmatocytes*, tumor apoptosis is also induced by TNF secreted by immune cells. In addition, hemocytes are recruited by activated JNK signaling in tumors. Mmp2, a target of the JNK pathway, causes basement membrane damage and recruits hemocytes associated with tumors (193). On the other hand, the JNK pathway plays a pro-tumorigenic role by promoting excessive proliferation and inflammatory reactions. Pinal et al. showed that in apoptosis-deficient cells, the JNK pathway is continuously activated by stress, which induces excessive cell proliferation and tumorigenicity (201). In addition, Zhou et al. showed that tumors caused by loss of BMP lead to JNK pathway activation and high Mmp2 expression levels. Mmp2 causes dysfunction of the intestinal barrier and commensal imbalance, which lead to inflammatory reactions. The inflammation-positive feedback activates the JNK pathway in tumors (**Figures 2B, C**) (194).

The JNK pathway is required for multiple wound healing processes; it is activated at the edge of a wound in *Drosophila* epithelial cells (20) and wing imaginal disc cells (202). Downregulation of JNK pathway components such as Hep, Bsk, Slpr and Jun in epithelial cells induces defects in wound healing (203, 204). The JNK pathway contributes to wound healing through epithelial cell migration, elimination of dying cells and cell fusion. A target of the JNK pathway, Mmp1, can degrade the extracellular matrix and induce the migration of epithelial cells at the edge of a wound (192, 205, 206). Another target of the JNK pathway, Profilin, can cause actin polymerization to enhance the migration of epithelial cells (207). Dying cells in wounds need to be extruded to enable regeneration. Iida et al. showed that dying cell elimination is mediated by the JNK pathway (208). In addition, the JNK pathway cooperates with the JAK/STAT pathway and spatiotemporally regulates cell fusion during wound healing. Lee et al. indicated that JNK pathway activity peaks in the vicinity of a wound approximately 8 h after injury to promote cell fusion. JAK/STAT activity peaks at a later stage in a concentric ring slightly farther away from the wound site to suppress cell fusion. Furthermore, the JAK/STAT pathway in cells in the vicinity of the wound is suppressed by activated JNK signaling (209). Spatiotemporal regulation of cell fusion depends on a delicate balance between the JNK and JAK/STAT pathways during wound healing (**Figures 2B, C**).

## Tissue Communication in Innate Immunity

**Figure 3:**
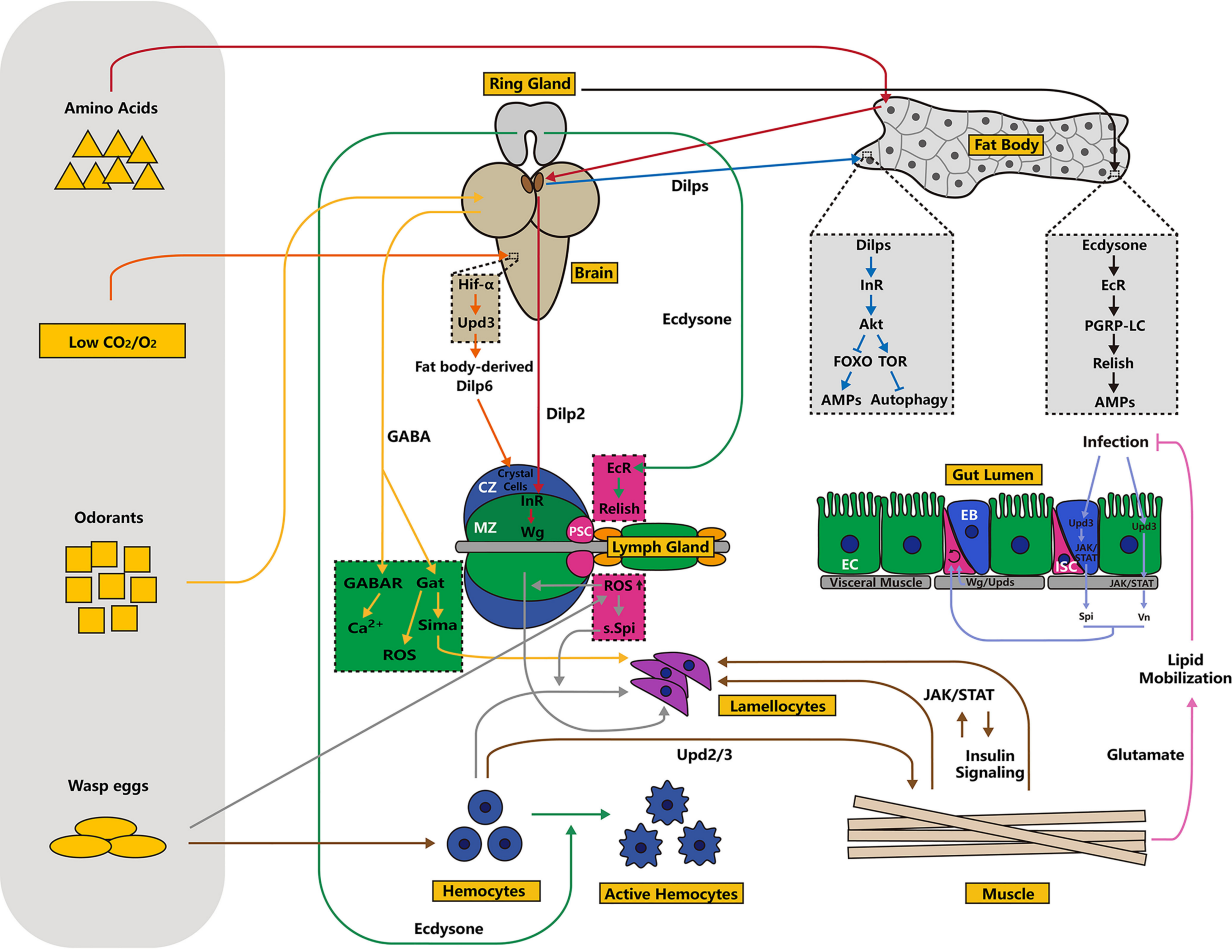
Schematic overview of tissue communication in innate immunity. In addition to autonomous regulation inside immune organs, homeostasis of the immune system is regulated by external signals emitted by other tissues, including the brain, ring glands, muscles and others. Tissue communication is largely dependent on two types of hormones: ecdysone and insulin. Moreover, ligands, including Upds, Vn and Spi, act as “messengers” in this process. Typically, the lymph gland is sensitive to nutrient status, oxygen concentration, odor signals and other environmental factors. Different processes or signaling pathways are indicated by arrows of different colors.

As described in the previous sections, the fat body, hemocytes and gut respond to immune challenge through intracellular signaling. However, the homeostasis of the immune system is maintained by signal transduction. Here, we will illustrate tissue communication in innate immunity by showing the functional roles of the brain and nervous system, muscles and the ring gland, which are largely dependent on hormonal regulation and multiple ligands (**Figure 3**). We also describe the potential regulatory role of the lymph gland in controlling hemocyte activation.

### The Brain and Nervous System

Similar to a central processing unit of the computer, the brain and nervous system control various biological processes, including development, metabolism and immune responses. In fact, the brain is immune-privileged and can respond to injury, infection and neurodegenerative disease through multiple innate immunity-related signaling pathways (210, 211). For instance, in the adult *Drosophila* brain, ZIKA virus induces the activation of the Imd signaling pathway. In this context, autophagy is activated through ubiquitinated proteins that bind to Ref(2)P. Together, upregulated AMPs and high autophagy levels suppress the replication of ZIKA virus (212).

Brain control of innate immunity partially depends on insulin signaling. In flies, insulin-like peptides (Dilps) are secreted by insulin-producing cells located in the brain (213, 214). Dlips bind the only insulin receptor that is orthologous to insulin receptors in vertebrates. The active insulin receptor then induces its downstream target, Akt, which negatively regulates thetranscription factor FOXO and positively regulates target of rapamycin (TOR) (**Figure 3**) (215, 216). FOXO can directly promote the production of AMPs through its interactions with the promoters of NF-κB-dependent target genes (**Figure 3**) (217). In addition, TOR signaling inhibits autophagy, which is required for innate immunity (**Figure 3**) (218). For instance, the bacterium *Listeria monocytogenes* is recognized by PGRP-LE in hemocytes and then cleared by autophagy mediated through Atg8 (219). Autophagy can clear pathogens, including *Mycobacterium marinum* and *Salmonella enterica* (220). These outcomes highlight the importance of metabolic signaling in innate immune responses. However, excessive immune activation can alter metabolism and inhibit fly growth, suggesting that metabolism and immunity systems involve complex interactions (221, 222).The insulin/TOR signaling axis plays dual roles in controlling *Drosophila* hematopoiesis. First, this axis regulates the number and activity of the PSC in the lymph gland in a cell-autonomous manner. Second, its function in the MZ controls the maintenance of blood cell progenitors (223, 224). Consistent with this outcome, a study showed that amino acid signals are recognized in the fat body, stimulating the brain to release Dilp2, which binds the insulin receptor in the MZ to promote progenitor maintenance *via* Wingless (Wg) signaling (**Figure 3**) (18). Similarly, low CO_2_ or O_2_ triggers the stabilization of hypoxia inducible factor-α (Hif-α) in neurons of the ventral nerve cord, which upregulates the expression of the cytokine *upd3* in the brain. Active cytokine activity triggers JAK/STAT pathway activation in fat bodies, promoting the release of Dilp6. This secreted protein activates the insulin receptor and induces crystal cell differentiation through Serrate, a ligand for Notch signaling (**Figure 3**) (225).

Olfaction is another regulatory factor required for the immune response. Upon olfaction stimulation, a small cluster of neurosecretory cells acting downstream of olfactory receptor neurons releases GABA into hemolymph and binds the GABA_B_ receptor in the MZ, leading to high cytosolic Ca^2+^ levels, which maintain blood cell progenitors in the lymph gland (**Figure 3**) (226). Olfaction-derived systemic GABA also maintains ROS homeostasis in hematopoietic progenitors, which is essential for lymph gland growth (**Figure 3**) (227). Moreover, odors emitted by wasps induce neurosecretory cell secretion of GABA, which is sensed by the GABA transporter (Gat) in the lymph gland MZ, resulting in the stabilization of Sima protein through intracellular catabolism. This process eventually primes lamellocyte differentiation and boosts immunity against wasp egg challenge (**Figure 3**) (228). A feeding behavior assay showed that adult flies are initially attracted by the odors emitted by pathogenic bacteria. However, the ingestion leads to long-term suppression of feeding behaviors. This process is mushroom body-dependent, and the octopaminergic neuron-derived immune receptors PGRP-LC and PGRP-LE are involved in this behavior (229). In summary, the olfaction/neuron axis is critical for flies to overcome immune challenges.

In *Drosophila* larvae, a subset of hemocytes consisting of sessile hemocytes is attached to the cuticle (16, 230). In fact, sessile hemocytes are not randomly distributed but cluster in every larval segment (109, 230). Sessile hemocytes are regarded as hematopoietic tissues because they can regulate blood cell differentiation (231). In addition, upon parasitoid wasp challenge, sessile hemocytes are released into circulation and can transdifferentiate into lamellocytes (232). Some studies have identified the peripheral nervous system as a microenvironment for sessile hemocyte homeostasis (230, 233). Activin-β (a TGF-β family ligand) in sensory neurons of the peripheral nervous system is required for sessile hemocyte adhesion and proliferation (233).

The aforementioned studies on sessile hemocytes strongly indicate that the brain and nervous system play important roles in regulating innate immunity in *Drosophila*. However, a series of studies have revealed that immune responses are also involved in neurodegenerative disease progression. For example, extracellular deposits of insoluble fibrillar amyloid-*β* (A*β*) peptides constitute the hallmark of Alzheimer’s disease (AD) (234, 235). In a *Drosophila* AD model, the JAK/STAT and JNK/AP-1 signaling pathways are activated and required for the clearance of neurotoxic A*β* peptides (236). In addition, Imd and JNK signaling are involved in a *Drosophila* model of Parkinson’s disease, and inhibition of *Rel* in dopaminergic neurons rescues mobility defects and neuronal loss (237). In summary, the *Drosophila* brain is not only an appropriate model for studying the systemic control of immune responses but is also useful for understanding the interaction between innate immunity and neurodegenerative diseases.

### Muscles

Muscles have been well characterized as tissues that control movement. However, a series of studies has highlighted the significant roles played by muscles in the innate immune response. For example, in *Drosophila*, indirect flight muscles produce AMPs upon bacterial challenge, which is essential for survival after infection (238). Yang and colleagues have identified the somatic muscle as a vital immunological tissue in the fight against parasitoid infection (239, 240). First, wasp eggs trigger the upregulation of JAK/STAT signaling ligands Upd2 and Upd3 in hemocytes and then activate the JAK/STAT signaling pathway in muscles, which is required for lamellocyte differentiation and the successful encapsulation of the infesting eggs (**Figure 3**) (239). In addition to the JAK/STAT pathway, muscle-derived insulin signaling has also been shown to be important for an efficient encapsulation response (**Figure 3**) (240). During this process, the JAK/STAT and insulin signaling pathways engage in reciprocal interactions (**Figure 3**). Insulin signaling positively regulates JAK/STAT signaling under normal and immune challenge conditions (**Figure 3**) (240). Moreover, activating insulin signaling rescues encapsulation rate and lamellocyte formation defects caused by suppressing JAK/STAT signaling in muscles, and vice versa (**Figure 3**) (240). These data indicate that muscles and hemocytes coordinate to mediate the cellular immune response upon wasp egg challenge. However, the mechanism underlying how muscle-derived signals convert prohemocytes/plasmatocytes into lamellocytes remains unclear. Inspired by the role played by NimB5, a secreted protein produced by the fat body during nutrient scarcity, in controlling circulating hemocyte count, we reason that metabolic processes affect lamellocyte activity, although the mechanisms warrant further research (241).

The JAK/STAT pathway in muscles systemically modulates insulin signaling. Muscle-derived JAK/STAT positively controls insulin signaling in fat bodies, and inhibition of JAK/STAT in muscles upregulates the transcription of *dilp2* and *dilp5* but downregulates *dilp3* transcription in larval brains (240). Consistent with these findings, another study showed that loss of the *dome* receptor in adult muscles significantly reduces fly lifespan and causes metabolic pathologies, and these outcomes are associated with Akt hyperactivation and subsequent metabolism dysregulation (242). Furthermore, glutamate in muscle promotes vitamin-dependent lipid mobilization in fat bodies and improves intestinal pathogen clearance, which eventually increases survival rates upon bacterial infection (243). These data confirm that somatic muscles integrate immune and metabolic signaling. Therefore, in addition to the fat body, muscle may be a promising model for determining how hosts balance metabolism and immune responses. Different somatic muscles whose functional roles in regulating immune responses are largely unknown, but visceral intestinal are known to control intestinal homeostasis and local immune responses. During the embryonic stage, visceral muscle-derived *frizzled 2* and *Wnt4*, which encode the receptor and ligand of canonical Wnt signaling, respectively, are required for left-right asymmetric development of the anterior midgut (244). Similarly, upon damage or bacterial infection, visceral muscles secret the Wnt pathway ligand Wg, regulating ISC maintenance and non-cell-autonomous proliferation (245, 246). In addition, Upd signals in visceral muscles activate JAK/STAT in ISCs to maintain ISC self-renewal and differentiation. Following infection, damaged enterocytes (ECs, gut epithelial cells) and enteroblasts (EBs, EC precursors) secret Upd3 to activate the JAK/STAT signaling pathway in visceral muscles and EBs, which trigger the secretion of two EGFR signaling ligands, Vein (Vn) and Spitz (Spi), from visceral muscles and EBs, respectively (**Figure 3**). Vn and Spi stimulate ISC proliferation through the EGFR signaling pathway (**Figure 3**) (247–250). Furthermore, a recent study showed that inhibition of *Pngl*, the homolog of human *N*-glycanase 1 (*NGLY1*), in the visceral muscles of the larval intestine significantly decreases AMP-activated protein kinase α (AMPKα) levels, disrupting gut homeostasis (251). Collectively, these studies validate visceral muscles as a niche in the control of intestinal homeostasis in a non-cell-autonomous manner. To test whether somatic muscles employ a similar signaling transduction mechanism as that identified in intestinal visceral muscles, Yang and Hultmark examined the transcript levels of EGFR ligands, Vn, Spi, Keren and Gurken, as well as Wnt4, during wasp egg infection. However, the expression of none of these genes was upregulated, indicating that different muscle types regulate immune responses in different patterns (252).

### The Ring Gland and Hormones

The ring gland is an endocrine organ that controls development, growth and reproduction in *Drosophila* by producing multiple hormones (253). The ring gland consists of three tissues: prothoracic gland, corpus allatum and corpora cardiaca tissues (254). The prothoracic gland is the largest part of the ring gland, and it secretes the molting hormone ecdysone, while the corpus allatumsynthesizes juvenile hormone (253–255). Early studies showed that ecdysone and juvenile hormone regulate AMP expression upon immune challenge (256). They confirm the role of ecdysone in mediating immune responses, showing that ecdysone controls the expression of the pattern recognition receptor PGRP-LC, subsequently modulating innate immune recognition and host defense against bacterial challenge (**Figure 3**) (257). In addition, ecdysone regulates the expression of *drs* and *drosomycin-like 2* (*drsl2*) systemically and locally in the midgut, respectively, and Drs induction depends on *Broad* (*Br*, an early ecdysone-response gene) (258). Regarding cellular immunity, ecdysone activates hemocytes by regulating both actin and the tubulin cytoskeleton (**Figure 3**) (259, 260). Moreover, hemocytes insensitive to ecdysone show impaired phagocytosis, and pupae with ecdysone-insensitive hemocytes show higher lethality upon septic and oral infection (259). Similar to humoral immunity, the activation of Rel in the hematopoietic niche of the lymph gland is also controlled by ecdysone signaling, and downregulation of ecdysone receptor (EcR) in the PSC results in excessive differentiation of plasmatocytes (**Figure 3**) (143). Because downregulated PSC-derived Rel expression boosts the immune response during bacterial infection, we speculate that ecdysone signaling plays an essential role in hematopoiesis under both normal and infectious conditions (143). These results highlight the importance of ecdysone signaling in mediating the cellular immune response. In summary, the ring gland systemically regulates innate immunity by secreting hormones, although the mechanism of this action warrants further investigation (**Figure 3**). Xiong et al. revealed that *microRNA-34* (*miR-34*) mediates both ecdysone signaling and innate immunity by acting as a node, suggesting that microRNAs might be key regulators in tissue communications (261, 262).

### The Lymph Gland

As described in the previous sections, the lymph gland, an immunological organ, not only participates in hematopoiesis but also responds to wasp egg challenge. In addition to its effect on nutrient and olfactory signaling, iron homeostasis controls blood cell differentiation in the lymph gland, as indicated by loss of *Fer1HCH* in the intestine causing an increase in the crystal cell count (263). However, the mechanism by which the lymph gland regulates other tissues remains unclear. Sinenko et al. proposed a model in which invading parasite eggs induce high levels of ROS production in the PSC, which in turn induces the secretion of Spi, which eventually results in the differentiation of lamellocyte precursors in the circulatory system mediated through the Ras/Erk pathway (**Figure 3**) (62). This speculation is based on lymph glands playing a regulatory role in controlling circulating hemocyte differentiation. A subsequent study indicated that high sSpi levels in the PSC caused by parasites activate the EGFR signaling pathway in the MZ in a non-cell-autonomous fashion, and this increase in EGFR signal transduction is required for lymph gland lamellocyte differentiation (63). Another study reported that repressing *headcase* (*hdc*) in the PSC induces lamellocyte differentiation in the hemolymph. Lineage-tracing assays suggested that the majority of lamellocytes are not derived from the lymph gland, confirming PSC to be a niche in which circulating hemocyte differentiation is regulated (264). Moreover, Khadilkar et al. highlighted the critical function of the lymph gland in regulating circulating hemocytes, showing that genetic ablation of occluding junctions in the PSC boosts the cellular immune response in the circulatory system (265). However, most hematopoietic system-specific *Gal4* drivers are expressed in hemocytes in both the circulatory system and the lymph gland, resulting in investigations into the communication within the blood system are difficult to discern. Knocking down *Arf1* expression only in circulating hemocytes with *Gcm-Gal4* induces excessive differentiation of crystal cells and plasmatocytes in the lymph gland (266). Thus, the development additional cell-specific genetic tools will enable us to understand how lymph glands and circulating hemocytes interact with each other and may reveal the functional role of the lymph gland in controlling the homeostasis of other tissues. Furthermore, whether hemocytes act as “messengers” in this process is of great interest and a topic for future study.

## *Drosophila* as a Model for Studying Innate Immunity

In mammals, the protein complex NF-κB is critical for innate immunity, while NF-κB induces the AMP production upon pathogen invasion in the fat body of *Drosophila*. In the fly genome, three genes, *rel*, *dorsal* and *Dif*, encode NF-κB-like proteins, which participate in the Imd and Toll signaling pathways (21, 267, 268). The Toll signaling ligand/cytokine Spz, a homolog of mammalian IL-17, has an active C-terminal region whose cysteine residues share similarities with those found in cysteine-knot growth factors (95, 269–271). In addition, this region forms dimers similar to those of vertebrate nerve growth factors (270, 272). In addition to Spz, the JAK/STAT ligand Upd3 is a homolog of the mammalian cytokine IL-6 (155, 180). Egr, a member of the TNF family, can activate the JNK pathway, consistent with the roles of mammalian TNFs (182, 183). Moreover, Wgn is the first TNF receptor homolog to be identified in flies (184). In summary, *Drosophila* shares evolutionarily conserved cytokines and regulatory factors in innate immunity with mammals.

Regarding cellular immunity, signaling pathways maintaining *Drosophila* hematopoietic homeostasis have been shown to play important roles in mammalian hematopoiesis. For instance, the lineage commitment of hemocytes is tightly regulated by GATA, Friend of GATA and RUNX factors, which are also key regulators in controlling hematopoiesis in mammals (273). In addition, the Wg/Wnt and JAK/STAT signaling pathways, which are critical for lymph gland progenitor maintenance, are crucial for hematopoietic stem cell renewal (274, 275). To date, two different mammalian hematopoietic niches have been identified: the endosteal niche and the perivascular niche (276). Suppressing BMP receptor 1A in mouse bone marrow stroma induces an increase in the osteoblast count, and the process is similar to that of BMP signaling pathway inhibition in the PSC of the lymph gland (277, 278). Furthermore, upon bacterial infection, neutrophils are produced *via* TLR (Toll-like Receptor)/NF-kB signaling and activated in mouse bone marrow endothelial cells, components of the vascular niche (279, 280). Similarly, wasp egg challenge elevates ROS levels in the PSC, promoting lamellocyte differentiation through the activation of EGFR and Toll signaling pathways (63).

Another reason that *Drosophila* is a useful model is based on the ability to perform tractable genetic manipulations. Through the *UAS/Gal4* system, time- and tissue-specific expression of certain genes can be achieved. Overexpression of AvrA, an effector protein in *Salmonella typhimurium*, in the fat body affects the proper activation of the Imd pathway (281, 282). However, acatalytically dead form of AvrA exerts no effects on Imd signaling, suggesting that AvrA enzyme activity plays a key role in regulating host immunity (282). Similarly, in the fat body, genetic activation of viral protein U (Vpu), an accessory protein in human immunodeficiency virus (HIV), inhibits Toll-dependent immune responses and impairs the ability of flies to combat fungal infection; this phenotype is similar to the fungal infection susceptibility phenotype observed in acquired immunodeficiency syndrome (AIDS) patients (131, 283). Another study suggested that Vpu induces apoptosis in *Drosophila* wings mediated through the JNK signaling pathway (284). Furthermore, using the *UAS/Gal4* system to ectopically express human AML-associated *NUP98-HOXA9* (*NA9*) induced leukemia-like phenotypes: excessive proliferation of blood cells and hyperplastic growth of the hematopoietic organ (285). Collectively, due to the parallels in signaling pathways and regulatory factors observed between flies and mammals and the convenience of the genetic manipulation in flies, *Drosophila* is an ideal model for studying both innate immunity and human disease pathology.

## Conclusions and Future Perspectives

In this review, we show that *Drosophila* relies on a powerful innate immune system to combat various invading pathogens. Upon immune challenge, a series of AMPs are produced in the fat body and released into hemolymph in a process known as the humoral immune response or systemic immune response. AMP synthesis is mediated by two NF-κB-related pathways: the Toll and Imd pathways. In addition, the JAK/STAT and JNK pathways play important roles in innate immune responses. The cellular immune response is another defense mechanism activated to fight foreign intruders, and three types of hemocytes are involved in this response: plasmatocytes, crystal cells and lamellocytes. Plasmatocytes, macrophage-like cells, kill pathogens through phagocytosis, and crystal cells participate in wound healing through melanization. Although lamellocytes are rare under normal conditions, wasp egg challenge induces their proliferation because they encapsulate large foreign bodies, such as wasp eggs. We also outline tissue communications in terms of innate immunity and show that the brain, muscles, the ring gland and the lymph gland maintain the homeostasis of the immunological system, largely through hormonal regulation and a series of cytokines. Because of the similarities between fly and mammalian innate immunity-related signaling pathways, *Drosophila* is a useful model for studying host-pathogen interactions. In addition, tractable genetic manipulation and convenient tools, such as the *UAS/Gal4* system, have allowed the use of *Drosophila* in investigations into infectious diseases.

As mentioned above, the mechanisms underlying pathogens recognition and clearance are relatively well understood. However, the communication between hemocytes and the fat body, as well as the relationship between humoral and cellular responses, is not very clear. Genomic, proteomic, and metabolomic analyses at the single-cell level using up-to-date technology are promising strategies to address issues of innate immunity in *Drosophila* (286, 287). In fact, in recent years, single-cell RNA sequencing and single-cell transcriptomics have been applied to studies on the *Drosophila* blood system. With the help of these technologies, novel clusters of hemocytes and lymph gland cells have been identified (39, 59, 288). Notably, Fu et al. characterized two previously unidentified *Drosophila* blood cell types: thanacytes and primocytes (40). Single-cell RNA sequencing has also led to the discovery of the novel role played by FGF in the immune response to parasitoid wasp eggs (39). Collectively, single-cell technologies have helped us better understand the complexity of the fly blood system and hemocytes differentiation and transdifferentiation upon immune challenge. In the future, the application of these technologies to studies on the fat body and other immune organs will likely reveal the heterogeneity of the cells in these tissues and previously unknown mechanism of tissue interactions in the immune context. Although studies on innate immunity have been performed at the single-cell level, an increasing number of studies have focused on the role played by AMPs in addition to those played in the immune responses. For instance, Diptericin B functions in the formation of long-term memory (289). AMPs have also been correlated with aging (290–292). Furthermore, a study illustrated that several AMP genes are upregulated in hematopoietic tumor-bearing larvae and that upregulated AMPs expression inhibits excess expansion of the lymph gland (54). Consistent with these findings, Dfn together with Eiger promotes tumor cell death, confirming an antitumor role played by AMPs (57). In summary, investigating the roles of innate immunity-related molecules in addition to those in the immune context is an interesting direction that will lead to insights into the coordination of the immune system with other biological processes, including metabolism, development and growth.

Finally, although *Drosophila* are believed to engage only innate immunity, increasing evidence shows that they may have the capacity for engaging adaptive immune-like responses. For example, pre-injecting flies with a sublethal dose of *Streptococcus pneumoniae* protects the flies from a second wave of infection (293). In line with this, immunological memory has been observed with hemocytes exposed to virus-derived short interfering RNA (siRNA)-containing exosomes, and antiviral immunological memory may be transmitted to progeny (294, 295). These studies encourage us to rethink adaptive immunity in flies. Because the viral proteins of severe acute respiratory syndrome coronavirus (SARS-CoV) have been studied in flies, *Drosophila* is a promising *in vivo* model for COVID-19-related research that may be used in the near future (296–298).

## Author Contributions

SY and FL contributed to the writing of this review article. YX and YZ contributed to the revision of this manuscript. LJ approved the final version of the manuscript. SY, FL, YX, YZ and LJ are accountable for the entire contents. All authors contributed to the article and approved the submitted version.

## Funding

This work was supported by the National Natural Science Foundation of China (32170484) and the Fundamental Research Funds for the Central Universities (2572022BD05).

## Conflict of Interest

The authors declare that the research was conducted in the absence of any commercial or financial relationships that could be construed as a potential conflict of interest.

## Publisher’s Note

All claims expressed in this article are solely those of the authors and do not necessarily represent those of their affiliated organizations, or those of the publisher, the editors and the reviewers. Any product that may be evaluated in this article, or claim that may be made by its manufacturer, is not guaranteed or endorsed by the publisher.
